# Histone Methyltransferase KMT2B Promotes Metastasis and Angiogenesis of Cervical Cancer by Upregulating EGF Expression

**DOI:** 10.7150/ijbs.72381

**Published:** 2023-01-01

**Authors:** Dan Zhao, Hui Yuan, Yuan Fang, Jian Gao, Huimin Li, Mengge Li, Hui Cong, Chenglin Zhang, Yiyi Liang, Jin Li, Hancao Yang, Ming Yao, Min Du, Hong Tu, Yu Gan

**Affiliations:** 1 State Key Laboratory of Oncogenes and Related Genes, Shanghai Cancer Institute, Renji Hospital, School of Medicine, Shanghai Jiao Tong University, Shanghai, China.; 2 Organ Transplantation Center, The First Affiliated Hospital of Kunming Medical University, 295 Xichang Road, Kunming, Yunnan Province, China.; 3 Department of Orthopedics, Changzheng Hospital, Naval Medical University, 415 Fengyang road, Shanghai, China.

**Keywords:** cervical cancer, KMT2B, angiogenesis, metastasis, EGF

## Abstract

Evidence has indicated that lysine methyltransferase 2B (KMT2B), a major H3K4 tri-methyltransferase (H3K4me3), contributes to the development of various cancers; however, its role in cervical cancer (CC) is unclear. In this study, increased KMT2B expression was observed in human CC specimens and significantly associated with poor prognosis. The condition medium of KMT2B-overexpressing cells facilitated angiogenesis* in vitro*. In the subcutaneous model of human CC, KMT2B overexpression significantly promoted tumor growth and increased tumor vascular density. Meanwhile, KMT2B enhanced the migration and invasion of CC cells and promoted their metastasis to bone in a tail-vein-metastasis model. Mechanistically, the genes upregulated by KMT2B were significantly enriched in PI3K-AKT pathway. Using H3K4me3 ChIP-seq analysis, we found increased H3K4me3 level at EGF promoter region in KMT2B-overexpressing HeLa cells. ChIP-qPCR experiments not only confirmed the increased H3K4me3 level of EGF promoter but also determined that in KMT2B-overexpressing HeLa cells, KMT2B increased binding with the EGF promoter. Blocking EGFR diminished the KMT2B-induced PI3K-AKT signaling activation and CC cell migration and invasion. Moreover, EGFR inhibitors abolished the KMT2B-drived tube formation capacity of HUVECs. In conclusion, KMT2B facilitates CC metastasis and angiogenesis by upregulating EGF expression, and may serve as a new therapeutic target for CC.

## Introduction

Cervical cancer (CC) ranked as the fourth most common cancer and the fourth cause of cancer mortality in women, with an estimated 604,000 new cases and 342,000 deaths worldwide in 2020 1. Thirteen percent of CC patients are diagnosed at advanced stages 2. For women with metastatic CC, especially bone metastasis, the overall prognosis is poor, resulting only 6 months survival in the absence of appropriate therapy 3.

Multiple epigenetic alterations, including global DNA hypomethylation, chromatin remodeling, as well as histone modifications, occur during whole stages of cervical carcinogenesis 4. Histone methylation is a common form of histone modification with demonstrated effects on gene transcription. So far, among six major histone lysine methylation sites 5, H3K4 is one of the most extensively studied methylation sites in histone methylation-mediated transcriptional regulations 5. Methylation of H3K4 with its mono-, di-, and tri-methylated forms are differentially enriched at promoter or enhancer sequence, contributing to the activation of gene expression 6.

H3K4 methylation is mainly catalyzed by the lysine methyltransferase 2 (KMT2) family (also known as mixed lineage leukemia (MLL) family), which contains six members (Set1a, Set1b, KMT2A, KMT2B, KMT2C, and KMT2D) 7. KMT2 proteins are found in three multimolecular complexes, each one containing either of two highly related paralogs as the catalytically active subunit: the Set1a-Set1b complex, the KMT2A-KMT2B complex, and the KMT2C-KMT2D complex 8. These complexes are responsible for H3K4 mono-, di-, tri-methylation 9, 10, in which KMT2B implement di- and tri-methylation at promoters and/or Polycomb response elements 11. KMT2 family is involved in various biological functions during the mammalian development 12. For instance, target deletion of both copies of KMT2A in mouse embryonic stem cells resulted in embryonic lethality 13. Knockout of KMT2B in germ-line resulted in delayed early development in embryogenesis and neural tube defects 14. Moreover, KMT2 proteins are also involved in a variety of pathological processes and their aberrant expressions are related to various diseases such as cancers 15.

In the field of cancer research, KMT2A, KMT2C and KMT2D have been intensively studied in different types of tumors 16-19. However, KMT2B is not studied as extensively and deeply as the other members. Unlike the well-established involvement of KMT2A translocations in acute leukemias, the implication of KMT2B, a close paralogue of KMT2A, was reported until recently. It was shown that KMT2B promoted proliferation of breast cancer cells through upregulating the expression of estrogen receptor alpha target genes 20. In colon cancer, KMT2B contributed to increased cancer stemness and decreased chemosensitivity via enhancing the expression of leucine-rich repeat containing G protein-coupled receptor 5 (LGR5), which is a composition of the Wnt receptor complex, by maintaining H3K4me3 at the promoter region of LGR5 gene 21. However, the role of KMT2B in CC has not been studied.

It is well established that the epidermal growth factor (EGF) contributes to a number of processes which are important to cancer development and progression 22. (EGF) is one of the most important mitogens for many epithelial cancer cells and typically exerts its biological function via binding to EGF receptor (EGFR) 23. High expression of EGFR was correlated with poor prognosis of CC patients 24. One of main intracellular signal transduction cascades downstream the activation of EGFR is phosphoinositide 3-kinase/protein kinase B (PI3K/AKT) pathway, which regulates cancer cell proliferation, migration and metabolism 25. In CC, the activation of EGF/PI3K/AKT signaling has been shown to promote tumor cells survival by inactivating several key apoptosis regulatory molecules 26. Also, EGF could enhance the migration and invasion of CC cells though PI3K/AKT signaling 27, 28. Blocking PI3K/AKT signal pathway by bacterial cyclodipeptides inhibited the proliferation and induced the apoptosis of CC cells 29, 30. Therefore, the EGF/PI3K/AKT pathway plays an important role in CC.

## Materials and Methods

### Tissue microarray and immunohistochemistry (IHC)

To analyze the KMT2B expression in CC tissues, a commercially available tissue microarray (National Engineering Center for Biochip, Shanghai, China) containing 30 pairs of CC tissues and the adjacent non-tumorous specimens was used for the IHC staining of KMT2B. The tissue samples were collected from Taizhou Hospital from 2006 to 2009 which were approved by Research Ethics Committee of Taizhou Hospital. The clinical characteristics of the tissue samples included in the tissue microarray are listed in **Table S1**.

The tumor tissues from nude mice with subcutaneous and myelogenous transplants tumors were resected, fixed in 10% neutral-buffered formalin, embedded in paraffin and sectioned at 5 μm. IHC was performed according to standard protocols. Briefly, the procedures for deparaffinization, rehydration, antigen retrieval and blocking were carried out as described previously 31. The sections were incubated with the primary antibodies at 4 °C overnight and treated with the secondary antibodies for 30 min at room temperature. The antibodies used for IHC are: anti-Ki67 (Cat. No. GB111141) and anti-CD31 (Cat. No. GB11063-3) from Servicebio Technology company (Wuhan, China). The intensity of the immunostaining was evaluated as follows: negative, 0 point; weak, 1 point; moderate, 2 points; strong positive, 3 points. The percentage of positive tumor cells was scored as 0 (<5%), 1 (<25%), 2 (25-50%), 3 (51-75%), and 4 (>75%). The final scores (0 to 12) were obtained by multiplying the two scores 32.

### Cell culture and treatment

The human CC cell line C-33A was obtained from the Cell Bank of the Chinese Academy of Sciences (Shanghai, China). The normal human cervical epithelial cell line HcerEpic cell was obtained from Mingzhou Biotechnology Co. (Ningbo, China). The Human Umbilical Vein Endothelial Cell line HUVEC and human CC cell line HeLa were purchased from the American Type Culture Collection (ATCC, Manassas, VA, USA). C-33A, HUVEC, and HeLa cells were maintained in DMEM medium supplemented with 10% FBS. HcerEpic cells were maintained in MEM medium supplemented with 10% FBS. All cells were cultured in a humidified atmosphere of 5% CO_2_ at 37 °C. For functional inhibition experiments, cells were treated with the PI3K inhibitor LY294002 (Cell Signaling Technology, Danvers, MA, USA) or the EGFR inhibitor gefitinib (Selleck Chemicals, Houston, TX, USA) at final concentrations of 20 μΜ or 200 nΜ, respectively.

### Public database

The mRNA expressions of KMT2B in CC tissues (n = 24), noncancerous cervical tissues (n = 28), and CC cell lines (n=9) were analyzed using the Gene Expression Omnibus (GEO) dataset GDS3233, which contains the largest sample size from the GEO (https://www.ncbi.nlm.nih.gov/geo/). We searched the GEO profiles of the gene KMT2B within the GDS3323 dataset and obtained expression values (normalized signal count values) from GEO profile data pages. This dataset was generated on the Affymetrix Human Genome U133A Array (HG-U133A) platform, which has 3 probe sets designed to detect KMT2B. According to the JetSet scoring method 33, we selected the most reliable probe set (203419_at) to represent KMT2B in our analysis. The overall and relapse-free survival curves of CC patients from the TCGA dataset were generated by the online survival analysis tool Kaplan-Meier plotter (http://kmplot.com/analysis/) 34. The KMT2B low expression and high expression groups were classified using the “Auto select best cutoff” function. The survival curves were calculated using the Kaplan-Meier method and analyzed by the log-rank Mantel-Cox test.

### Gene overexpression and knockdown

The KMT2B-overexpressing CC cells (HeLa-KMT2B and C33A-KMT2B cells) were conducted by GeneChem Corporation (Shanghai, China) using a modified version of the CRISPR/dCas9 synergistic activation mediator (SAM) system 35. The CC cells infected with an empty viral vector (HeLa-VC and C-33A-VC) were used as control cells.

KMT2B was knocked out by the CRISPR/Cas9 gene editing system to conduct the HeLa-KMT2BKO and C-33A-KMT2BKO cells. The pSpCas9(BB)-2A-GFP (PX458) was a gift from Feng Zhang (Addgene plasmid #48138; http://n2t.net/addgene:48138; RRID:Addgene_48138) 36. According to Feng Zhang lab's protocol, the intended sgRNA oligos targeting KMT2B coding region were cloned into the pSpCas9(BB)-2A-GFP (PX458) vector, which utilized U6 promoter to drive sgRNA expression and utilized CBh promoter (an alternative version of CMV immediate enhancer/β-actin promoter) to drive the equal expression of Cas9 and GFP linked by a 2A self-cleaving peptide. The sgRNA sequences are described in **Table S2**. Then, 1μg of plasmids were transfected into C-33A and HeLa cells via electroporation using NucleofectorTM 2b Device (Lonza, #AAB-1001). The control HeLa (HeLa-SC) and C-33A (C-33A-SC) cells were transfected with scrambled sgRNA plasmids. At day 3 post electroporation, GFP positive cells were sorted using flow cytometry. The cutting efficiency was determined by T7EI assay in sorted cells. After 2 weeks of cell culture, the knockdown of KMT2B in sorted cells was confirmed by western blot analysis.

### Animal experiments

The animal studies were approved by the Animal Care and Use Committees of Shanghai Cancer Institute. To construct a subcutaneous CC model, HeLa-VC and HeLa-KMT2B cells (3 × 10^6^) were injected into the right flanks of five-week-old female BALB/c nude mice (n = 5). These nude mice have a genetic mutation that cause a deteriorated or absent thymus, resulting in an immunecomprised phenotype due to a greatly reduced number of T lymphocytes. Tumor volumes were measured at the indicated time intervals. Tumor volume was calculated using the equation volume = 0.5 × length × width^2^. The mice were sacrificed when the tumor size reached approximately 1 cm^3^ and the tumors were excised and weighed.

HeLa cells were transduced with a lentiviral vector containing the GFP-P2A-luciferase cassette which was purchased from GeneChem Corporation (Shanghai, China) to generate HeLa cells expressing luciferase (HeLa-luc cells). Then KMT2B-overexpressing and control HeLa-luc cells (HeLa-KMT2B-luc and HeLa-VC-luc cells) was conducted by SAM system as previous described in 2.4. For the metastasis model, 3×10^6^ (in 150 μl PBS) of the HeLa-VC-luc and HeLa-KMT2B-luc cells were injected through tail vein of female BALB/c nude mice (6 weeks old, n = 6). *In vivo* imaging system (IVIS) (Berthold Technologies, Bad Wildbad, Germany) was used to monitor the development of tumors every week from day 21 after tumor cell injection. Micro-computed tomograpgy scanner (Skyscan 1076, Kontich, Belgium) was used to confirm the bone metastases in the limbs. A mice model of CC tumor intraosseous growth was established by intra-tibia injection of 1×10^6^ (in 20 μl PBS) HeLa-VC-luc and HeLa-KMT2B-luc cells (n = 8). After two weeks of tumor growth, IVIS was used to quantitated photon flux to monitor the tumor growth in bone marrow.

### Western blot analysis

Western blot analysis was performed according to the procedure described previously 37. The antibodies used for western blotting are: anti-KMT2B (Cat. No. 63735), anti-GAPDH (Cat. No. 51332), anti-Histone H3 (Cat. No.4499), anti-p-PI3K (Cat. No. 4228) anti-PI3K (Cat. No.4257), anti-p-EGFR (Cat. No. 3777) and anti-p-AKT (Cat. No. 4060) from Cell Signaling Technology (Boston, USA). Anti-p-PI3K (Cat. No. 11508), anti-PI3K (Cat. No. 48184) and anti-AKT (Cat. No. 33748) from Signalway Antibody (Maryland, USA). Anti-Histone H3 (tri methyl K4) (Cat. No. ab8085) from Abcam (MA, USA). Anti-EGFR (Cat. No. ET1603-37) from Huaan Biotechnology Company (Hangzhou, China). All western blottings were quantified using ImageJ software and the quantitative results were presented as the relative expression levels of target proteins normalized to the corresponding loading controls or pan-protein levels.

### RNA-seq and bioinformatics analysis

Total RNA from HeLa-VC and HeLa-KMT2B cells was isolated using TRIzol reagent (Life Technologies, Rockville, MD, USA) according to the manufacturer's instructions, and subjected to NovelBio lab (Shanghai, China) for RNA-seq. Briefly, cDNA library sequencing was performed using an Illumina, HiseqXTen platform. High-quality reads were aligned to the human reference genome (GRCh38 NCBI) using hisat2. Gene expression was calculated from fragments per kilobase of transcript per million (FPKM) by expectation maximization (RSEM). The filter criteria of *P* ≤ 0.05 and fold-change ≥ 2 or ≤ 0.5 were used to determine the differential expression of genes 38. Pathway enrichment analysis of differentially expressed genes was performed according to KEGG database. The transcript profiles were deposited in the National Center for Biotechnology Information GEO database under the accession number GSE209928 (accession link: https://www.ncbi.nlm.nih.gov/geo/query/acc.cgi?acc=GSE209928).

### Quantitative real-time PCR

RNA isolation from CC cells and quantitative real-time PCR (qPCR) analysis of KMT2B and EGF genes were performed according to our previous description 37. The relative expression of the target genes was normalized to GAPDH. The primer sequences are provided in **Table S3**. Relative mRNA expression levels of genes were analyzed by the 2(-ΔΔCt) method 39.

### Chromatin Immunoprecipitation sequencing (ChIP-seq) and Chromatin Immunoprecipitation quantitative real-time PCR (ChIP-qPCR)

ChIP assay was performed as previously described 40 with slight modifications. For cross-linking and cell harvesting, 1.6×10^7^ cells in four 10 cm dishes were crosslinked in freshly prepared formaldehyde solution (1% final concentration) and then quenched with 125 mM glycine. For sonication, pellets were resuspended into microcentrifuge tubes and then fragmented with a Bioruptor Pico (Diagenode, Belgium) sonicator at 4 °C. Protein G Dynabeads (Cell Signaling Technology, MA, USA) were added to the chromatin lysate with the antibody of H3K4me3 (Abcam, Cambridge, UK) or KMT2B (Cell Signaling Technology, MA, USA) and incubated overnight at 4 °C. ChIP-DNA was purified by a standard protocol using the QIAquick Gel Extraction Kit (QIAGEN, Hilden, Germany).

For ChIP-qPCR experiments, Immunoprecipitated DNA was analyzed by q-PCR. The amount of DNA fragment co-precipitated with antibody was calculated and compared with the amount of the same genomic fragment in total input DNA, resulting in percentage of input. Primers for ChIP-qPCR are listed in **Table S3**.

For ChIP-seq, the ChIP assay was performed using anti-H3K4me3 antibody as described above. ChIP-seq library preparation and sequencing were performed by RiboBio (Guangzhou, China). Briefly, the eluted ChIP DNA was used to construct the libraries and then sequenced on Illumina HiSeq 2500. Reads were mapped to hg19 human genome using Bowtie2 software 38. The mapped reads which needed compared were then selected for further analysis.

### Cell migration and invasion assays

For the cell migration studies, CC cells (5 × 10^4^ cells/insert) were suspended in serum-free medium and plated in the top chamber of the non-coated culture inserts (Corning, NY, USA) 41. CC cells (1 × 10^5^ cells/insert) were plated in the top chamber of the Matrigel (Corning, NY, USA)-coated inserts for the cell invasion assay 42. The lower chamber contained medium with 10% FBS. After 8 or 12h of incubation, the cells on the lower surface of the membrane were stained with crystal violet. The cell migration or invasion ability was quantitated by counting the number of crystal violet-stained cells. In some of the experiments, the cells were pretreated with PI3K or EGFR inhibitor for 16 h, and then subjected to transwell migration or invasion assay 42.

### Endothelial tube formation assay

HUVECs were resuspended in condition medium of KMT2B-overexpressing and control HeLa or C-33A cells. Then the cell suspension was added in 96-well plates coated with 50 μl of Matrigel (Corning, NY, USA) at a concentration of 2 × 10^4^ cells per well. Tubules were photographed through microscopy and computed by Image Pro Plus software after incubating for 4 h at 37 °C with 5% CO_2_.

### Enzyme-linked immunosorbent assay (ELISA)

The EGF levels in the cell lysate prepared from HeLa-VC or HeLa-KMT2B cells were quantitated using a human EGF ELISA kit (Boster, Wuhan, China). Briefly, cells (4 × 10^6^) were detached and transferred to microfuge tubes. The tubes were centrifuged to pellet the cells. Lysis buffer (RayBiotech, Norcross, GA, USA) were added to lyse cells. The cell lysate was collected and detected immediately according to the EGF ELISA kit instruction manual.

### Dual luciferase reporter assay

The human EGF promoter regions spanning nucleotides -1022 to 214 (relative to the transcription initiation site) were amplified by PCR from HeLa cells and cloned into the* Nhe*I/*Xho*I sites of pGL3-basic vector (Promega, Madison, WI, USA) to generate EGF promoter luciferase reporter (pGL3-EGF). HeLa-VC cells or HeLa-KMT2B cells (1× 10^6^) were co-transfected with pGL3-EGF (2 µg) and pRL-TK Renilla luciferase expression plasmid (2 µg) via electroporation using NucleofectorTM 2b Device (Lonza). At 48 h post-electroporation, firefly and Renilla luciferase signals were determined with a Dual Luciferase Reporter Assay Kit (Promega).

### Statistical analysis

The statistical analysis was performed using GraphPad Prism (GraphPad Software, San Diego, CA, USA) and presented as mean ± SD. The significance of the differences between groups was assessed using the student's *t* test (for the normally distributed variables) or the Wilcoxon rank sum test (for the non-normally distributed variables). The level of significance was set at 0.05 for all analyses.

## Results

### KMT2B is upregulated in CC associated with prognosis of the disease

**Figure 1:**
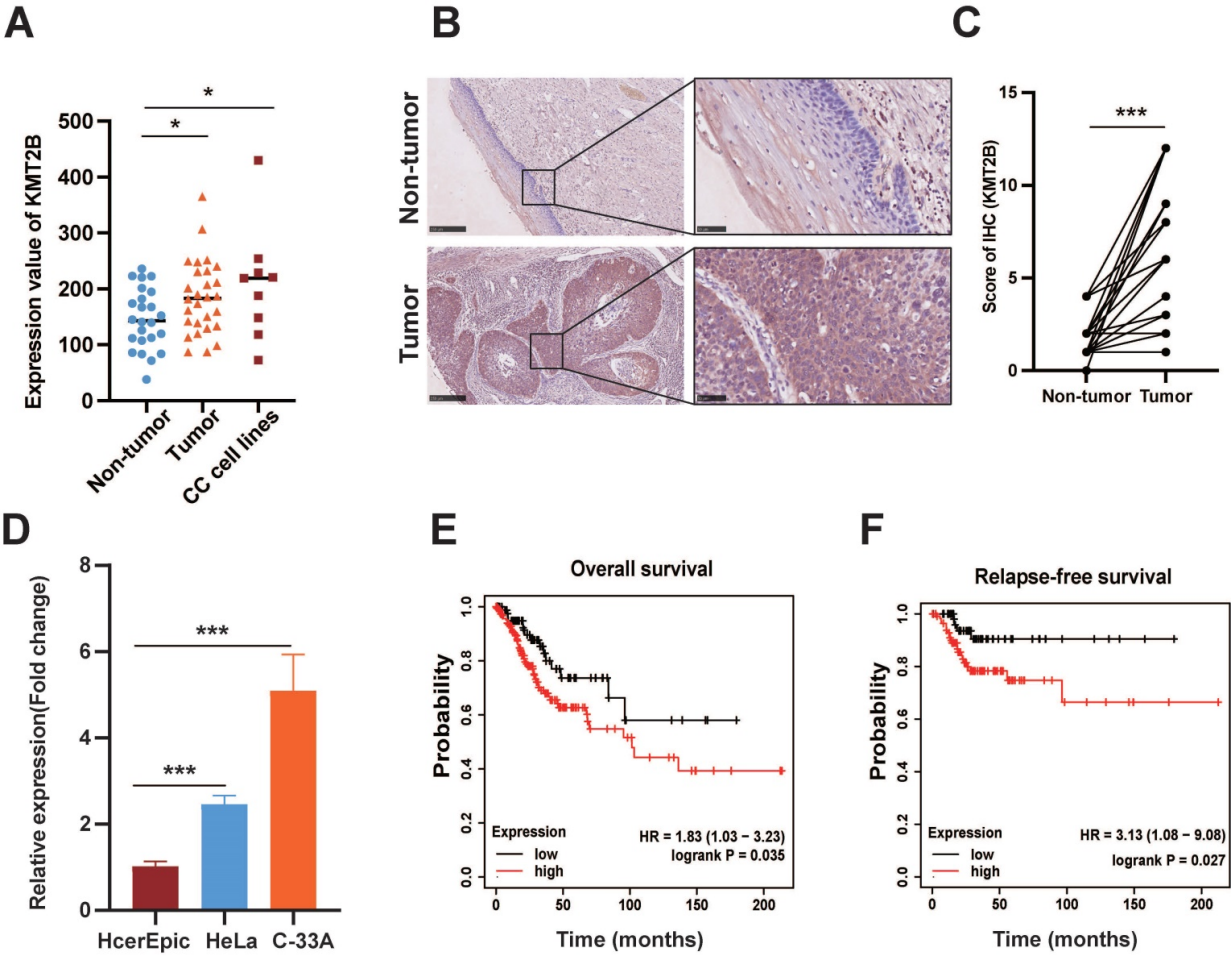
** KMT2B is upregulated in CC and associated with poor prognosis. (A)** The mRNA levels of KMT2B in CC (n = 24), adjacent non-tumor tissues (n = 28), and 9 CC cell lines obtained from the GEO profile. **(B)** Representative IHC images of KMT2B in CC tissues and adjacent nontumor cervical tissues. Scale bars, 10×, 250 µm; 40×, 50 µm; **(C)** IHC scores of KMT2B staining in CC tissues and matched adjacent nontumor tissues (n = 30). **(D)** Relative mRNA expression of KMT2B in CC cells (HeLa and C-33A) and normal cervical cells (HcerEpic) measured by qPCR (n = 3). The overall survival analysis **(E)** and relapse-free survival analysis **(F)** of CC patients based on the data from TCGA database using the web-accessible Kaplan-Meier plotter. Survival curves were calculated using the Kaplan-Meier method and analyzed by the log-rank test. *,* p* < 0.05, **, *p* < 0.01, ***, *p* < 0.001.

Based on the microarray-based gene expression profiles (GDS3233) with the largest sample size from GEO database, we found that the KMT2B mRNA expression was remarkably increased in CC tissues (n = 28) compared with noncancerous cervical tissues (n = 24). Meanwhile, CC cell lines also had a significantly higher level of KMT2B than noncancerous cervical tissues (both *p* < 0.05, **Fig. 1A**). In parallelly, we examined the protein expressions of KMT2B by immunohistochemical staining on tissue microarray (n = 30). Consistently, the protein levels of KMT2B were remarkably increased in CC tissues compared with adjacent noncancerous tissues (*p* < 0.001,** Fig. 1B-C**). As expected, two CC cell lines, namely HeLa and C-33A, exhibited significantly higher mRNA level of KMT2B than the normal cervical epithelial HcerEpic cells (*p* < 0.001,** Fig. 1D**). Furthermore, the survival analysis based on gene expression profiling and clinical data from TCGA database indicated that the patients with high KMT2B expression have significantly shorter overall survival [HR = 1.83 (1.03-3.23), *p* = 0.035] and relapse-free survival [HR = 3.13 (1.08-9.08), *p* = 0.027] compared with those carrying low KMT2B expression (**Fig. 1E-F**). The collective results indicated a potential role of KMT2B in CC progression.

### KMT2B promotes CC migration, invasion and angiogenesis *in vitro*

**Figure 2:**
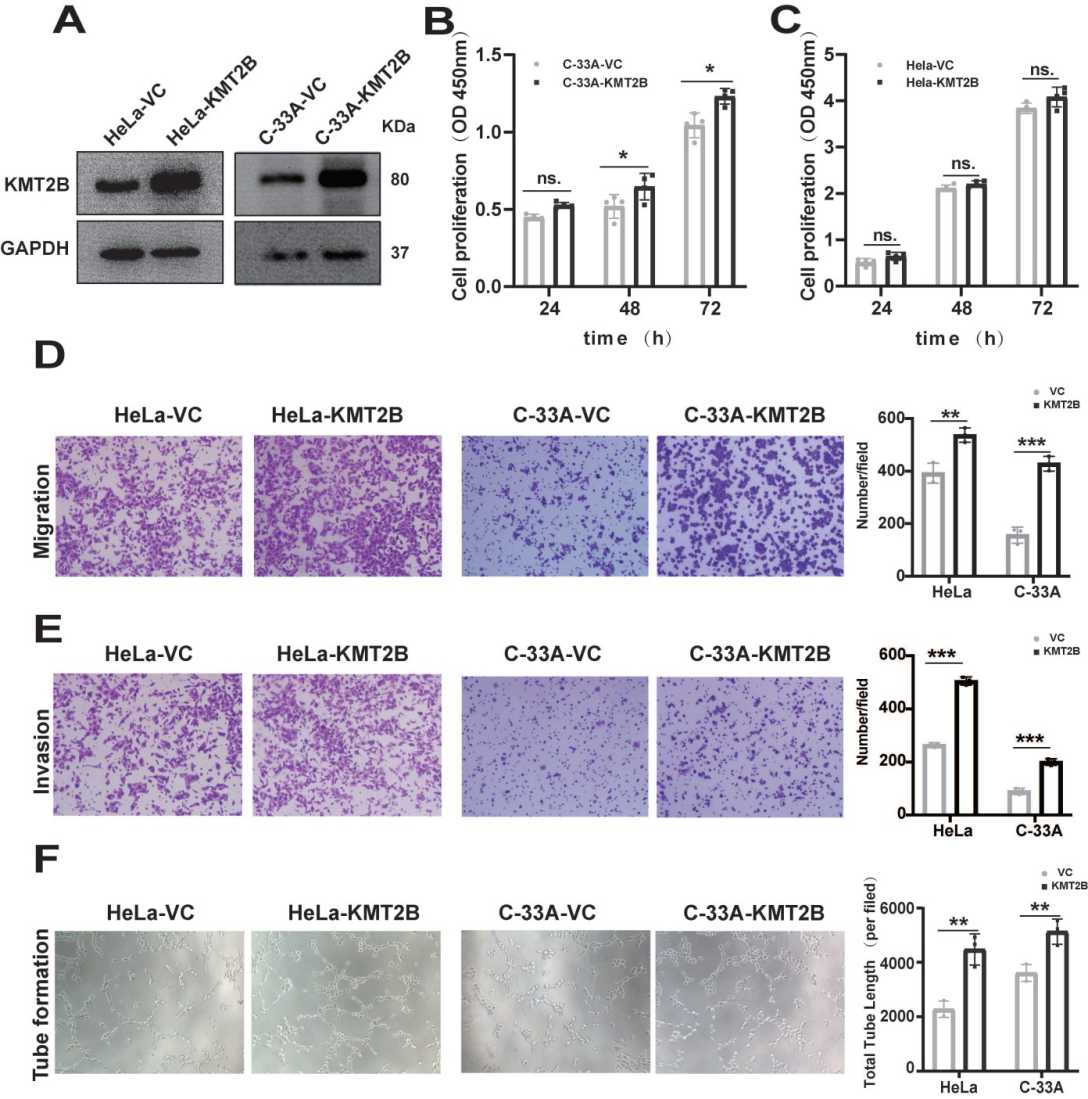
** Overexpression of KMT2B promotes CC cells growth, migration, invasion and angiogenesis*.* (A)** The protein levels of KMT2B in HeLa and C-33A cells after overexpressed by SAM system. **(B)** Cell proliferation of C-33A-VC and C-33A-KMT2B cells were measured with CCK8. **(C)** Cell proliferation of HeLa-VC and HeLa-KMT2B cells were measured with CCK8. **(D)** KMT2B-overexpressing and control HeLa or C-33A cells were cultured in non-coated chambers for 8 h. The cells that migrated through the chamber were stained using crystal violet and photographed. Quantitated the cells migration by counting the migrated cells. **(E)** KMT2B-overexpressing and control HeLa or C-33A cells were cultured in Matrigel-coated chambers for 12 h. The cells that invaded through the Matrigel were stained using crystal violet and photographed. Quantitated the cells invasion by counting the invaded cells. **(F)** Images of HUVECs tube formation with incubating in KMT2B-overexpressing and control HeLa or C-33A cells condition medium. Quantitated the total length of tubes by Image Pro Plus software after HUVECs incubating in KMT2B-overexpressing and control HeLa or C-33A cells condition medium for 4h. Data represents mean ± SD (n = 3). All the experiments have been repeated at least once with similar results. Statistical significance was determined by students* t* test. *, *p* < 0.05, **, *p* < 0.01, ***, *p* < 0.001, ns, not significant.

To investigate the functional role of KMT2B, we overexpressed KMT2B in human CC cell line HeLa and C-33A using a CRISPR-dCas9-SAM system. The overexpression of KMT2B, which was confirmed by qRT-PCR (*p* < 0.01, **Fig. S1A-B**) and Western blot (**Fig. 2A**), slightly promoted the proliferation in C-33A cells (*p* < 0.05,** Fig. 2B**) but not in HeLa cells (**Fig. 2C**). However, KMT2B overexpression significantly increased the migration abilities of both HeLa cells (*p* < 0.01,** Fig. 2D**) and C-33A cells (*p* < 0.001,** Fig. 2D**) by about 35% and 63.5%, respectively. KMT2B overexpression remarkably enhanced the invasion abilities of both HeLa cells (*p* < 0.001,** Fig. 2E**) and C-33A cells (*p* < 0.05,** Fig. 2E**) by about 91% and 93%, respectively. We also investigated the effect of KMT2B on CC-associated angiogenesis using an *in vitro* tube formation assay. HUVECs were incubated with the conditioned medium collected from CC cells and the results showed that the conditioned medium from KMT2B-overexpressing HeLa and C-33A cells significantly resulted in increased capillary tube formation by about 52% and 42%, respectively (*p* < 0.01,** Fig. 2F**), suggesting KMT2B promoted the migration, invasion, and pro-angiogenetic abilities of CC cells.

**Figure 3:**
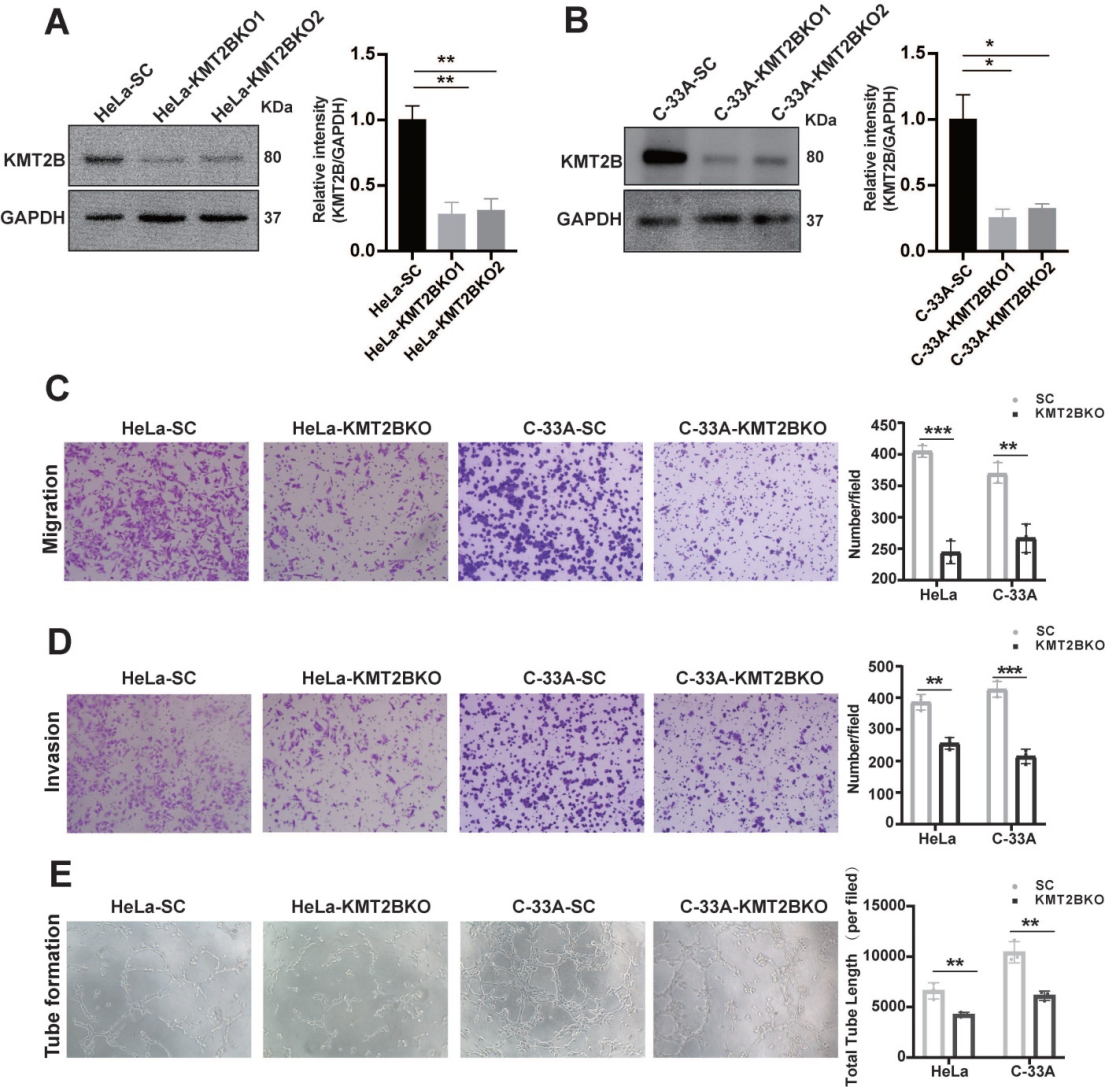
** The knockdown of KMT2B expression impairs CC migration, invasion and angiogenesis *in vitro*. (A, B)** The protein levels of KMT2B in HeLa and C-33A cells after knockdown by CRISPR/Cas9 method. The histogram shows the densitometric analysis of the bands. We selected the first sgRNA sequence with the highest knockdown efficiency for subsequent functional experiments. **(C)** KMT2B-knockdown and control HeLa or C-33A cells were cultured in non-coated chambers for 8 h. The cells that migrated through the chamber were stained using crystal violet and photographed. Quantitated the cells migration by counting the migrated cells. **(D)** KMT2B-knockdown and control HeLa or C-33A cells were cultured in Matrigel-coated chambers for 12 h. The cells that invaded through the Matrigel were stained using crystal violet and photographed. Quantitated the cells invasion by counting the invaded cells. **(E)** Images of HUVECs tube formation with incubating in KMT2B-knockdown and control HeLa or C-33A cells condition medium. Quantitated total length of tubes by Image Pro Plus software after incubating for 4 h in KMT2B-knockdown and control HeLa or C-33A groups. Data represents mean ± SD (n = 3). All the experiments have been repeated at least once with similar results. Statistical significance was determined by students* t* test. *, *p* < 0.05 **, *p* < 0.01; ***, *p* < 0.001.

To further verify the role of KMT2B in regulating the malignant behaviors of CC cells, we knock down KMT2B using CRISPR/Cas9 system in HeLa (*p* < 0.001, **Fig. 3A**) and C-33A cells (*p* < 0.001,** Fig. 3B**). Between the two sgRNA which specially targeted to the KMT2B gene, sgRNA1 reduced about 72% KMT2B expression in HeLa cells and 74.5% in C-33A cells and sgRNA2 reduced about 69% and 67.5% KMT2B expression in HeLa and C-33A cells. Therefore, the knockdown of KMT2B CC cells in subsequent experiments was constructed using sgRNA1. In contrast to the effect of KMT2B overexpression, the knockdown of KMT2B significantly suppressed the migration, invasion and pro-angiogenic abilities of HeLa and C-33A cells (*p* < 0.01,** Fig. 3C-E**).

### KMT2B promotes CC growth, angiogenesis, and bone metastasis* in vivo*

**Figure 4:**
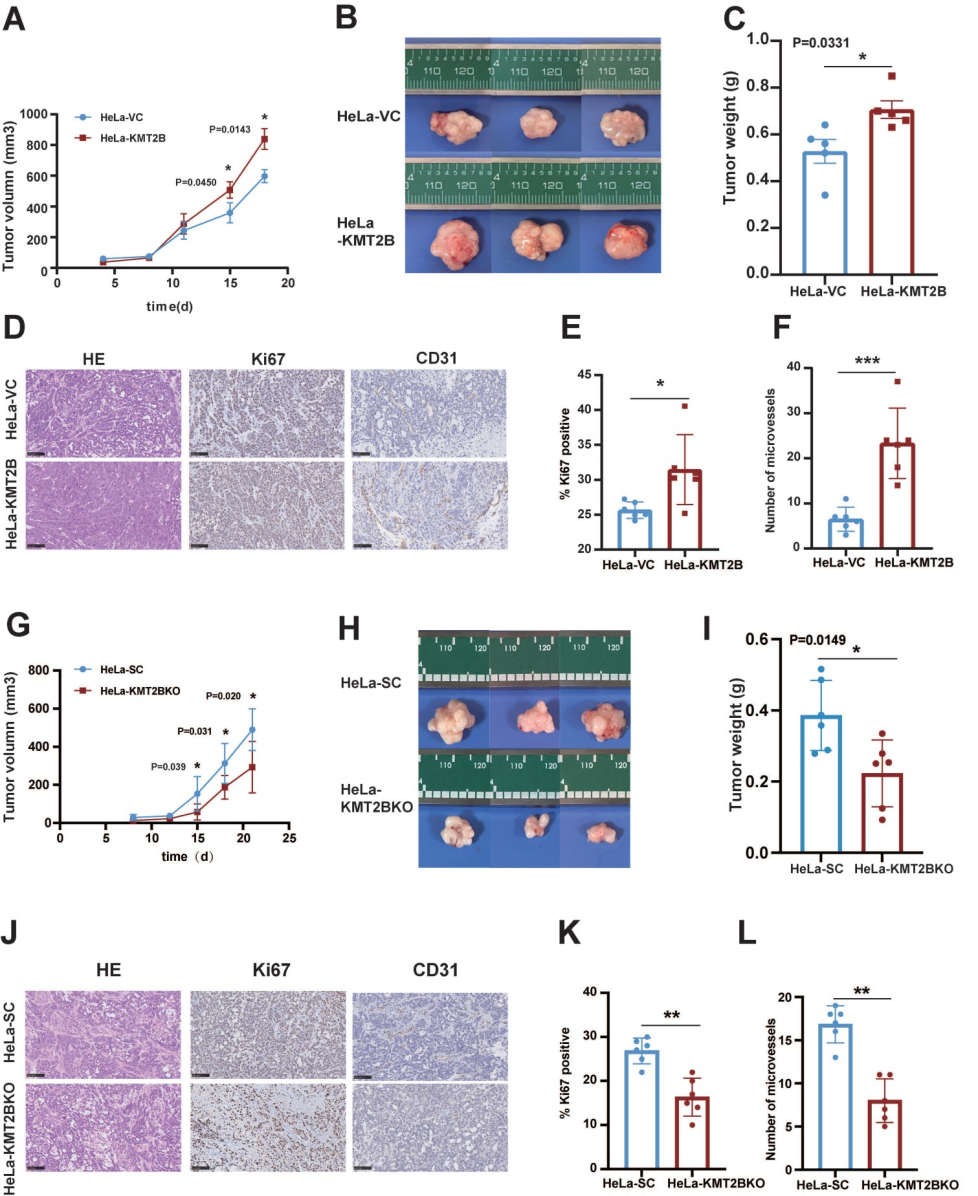
** KMT2B promotes CC growth and angiogenesis* in vivo.* (A)** Tumor growth curves of HeLa-VC and HeLa-KMT2B groups. The volumes of subcutaneous xenografts were measured during an 18-day period after tumor cell inoculation. (n = 5 for each group). **(B)** Representative images of subcutaneous xenografts tumors formed by HeLa-VC and HeLa-KMT2B cells. **(C)** Tumor weights of each group. **(D)** The representative hematoxylin-eosin (H&E) staining and the representative immunohistochemical staining of Ki67 and CD-31 of the subcutaneous tumors. Scale bars, 100 µm. **(E)** IHC analysis for Ki-67 expression was performed in tumors harvested from xenografts, and percents of the Ki-67 positive cells were quantified. (n = 6 for each group). **(F)** Number of microvessels was counted in each group. (n = 6 for each group). **(G)** Tumor growth curves of HeLa-SC and HeLa-KMT2BKO groups. The volumes of subcutaneous xenografts were measured during a 21-day period after tumor cell inoculation. (n = 6 for each group). **(H)** Representative images of subcutaneous xenografts tumors formed by HeLa-SC and HeLa-KMT2BKO cells. **(I)** Tumor weights of each group. **(J)** The representative H&E staining and the representative immunohistochemical staining of Ki67 and CD-31 of the subcutaneous tumors. Scale bars, 100 µm. **(K)** IHC analysis for Ki-67 expression was performed in tumors harvested from xenografts, and percent of the Ki-67 positive cells were quantified. (n = 6 for each group). **(L)** Number of microvessels was counted in each group. (n = 6 for each group). Data represent the mean ± SD. Statistical significance was determined by student's *t* test. *, *p* < 0.05, **,* p* < 0.01.

To investigate the* in vivo* role of KMT2B, HeLa-VC and HeLa-KMT2B cells were subcutaneously inoculated into athymic nude mice and the growth of tumors were monitored. Tumor growth was similar between the two groups during the first 8 days. Tumor growth was accelerated in both groups but the growth rate was significantly increased in the KMT2B-overexpressing group compared to the control group after 15 days (*p* < 0.05,** Fig. 4A**). The tumors were harvested and weighed at the time of sacrifice (18 days post-inoculation) (**Fig. 4B, C**). Consistent with the results of tumor growth, a significant increase in tumor weight was observed in KMT2B overexpression group compared to control group (0.71 ± 0.096 g *vs.* 0.52 ± 0.097 g, *p* = 0.0109), which was associated with markedly elevated Ki67 protein expression in KMT2B-overexpressing tumors (*p* < 0.05, **Fig. 4D-E**). A significant increase in the number of blood vessels (*p* < 0.001) was detected in KMT2B-overexpressing tumors by immunohistochemical staining of the endothelial cells by using the marker CD31 (**Fig. 4D, F**). In contrast, the knockdown of KMT2B significantly decreased the growth rate after 15 days of tumor growth, resulting in a 43% reduction in tumor weight (0.22 ± 0.085 g vs. 0.39 ± 0.089 g, *p* < 0.05) at the time of sacrifice (21 days post-inoculation) (**Fig. 4G-I**). We also observed a significant decrease in the percentage of proliferating tumor cells (*p* < 0.01) and the number of blood vessels (*p* < 0.001) in KMT2B-knockdown tumors as determined by immunohistochemical staining for Ki67 and CD31 (**Fig. 4J-L**). These results indicated that KMT2B could accelerate tumor growth and promote angiogenesis* in vivo*.

**Figure 5:**
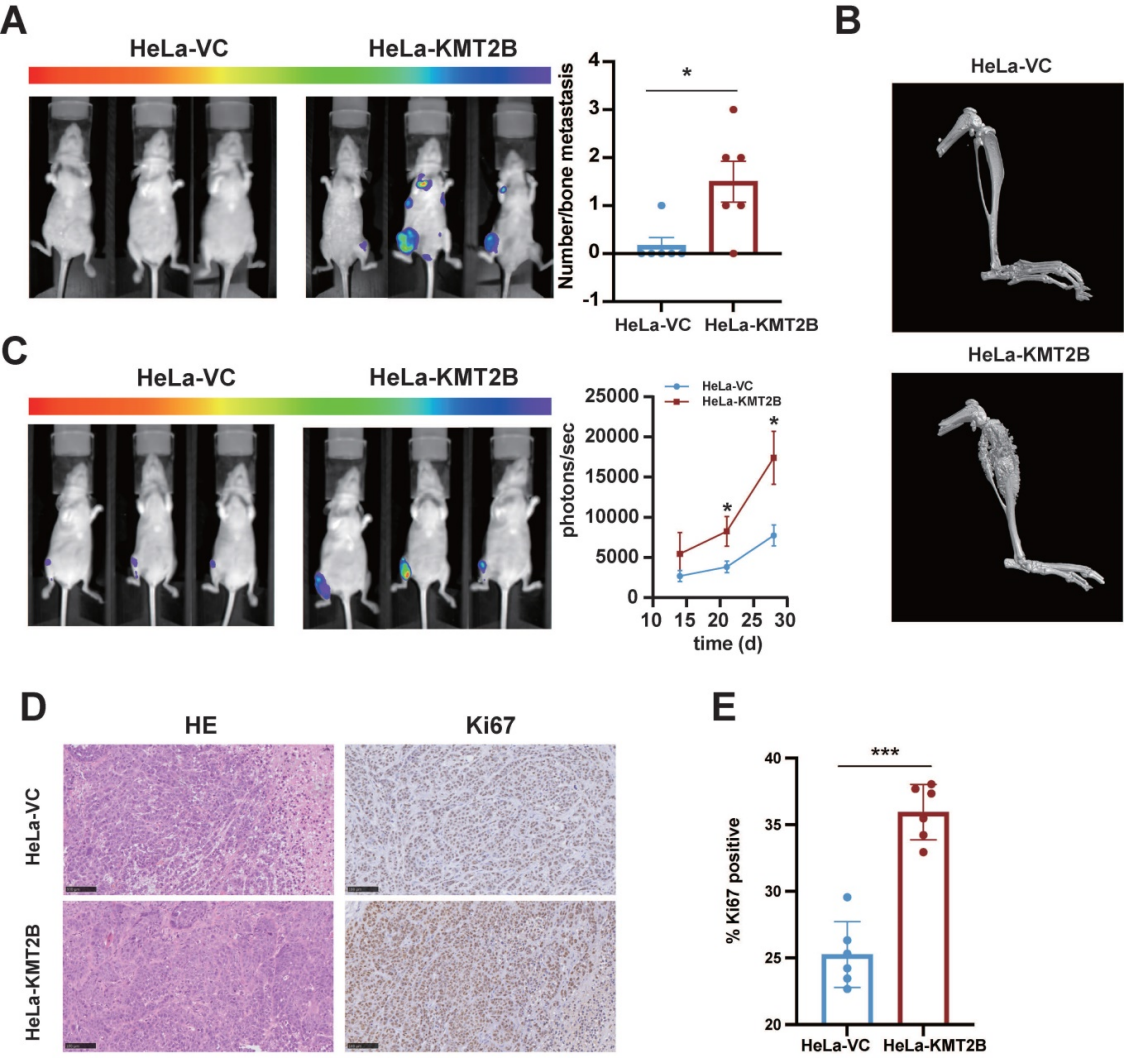
** KMT2B promotes CC bone metastasis* in vivo.* (A)** After 50 days of tail-vein injection of HeLa-VC-luc and HeLa-KMT2B-luc cells, the luciferase substrate was intraperitoneally injected, and chemiluminescence images were obtained and quantitated using IVIS. (n = 6 for each group). **(B)** Three-dimensional reconstruction images of osteogenesis metastasis in the limbs after tail vein injection of HeLa-VC-luc and HeLa-KMT2B-luc cells. **(C)** Bioluminescence was performed 14 days after tibia injection of HeLa-VC-luc and HeLa-KMT2B-luc cells every week. IVIS quantitated photon flux showed tumor growth in the bone marrow (n = 8 for each group). **(D)** Representative H&E staining and immunohistochemical staining of Ki67 in the myelogenous xenografts from both HeLa-VC and HeLa-KMT2B groups. Scale bars, 10×, 250 µm; 40×, 50 µm; **(E)** Quantitative analysis of Ki67^+^ cells (n = 6 for each group). Data represent the mean ± SD. Statistical significance was determined by student's* t* test. *, *p* < 0.05, **, *p* < 0.01, ***, *p* < 0.001.

We next tested whether KMT2B promote CC metastasis *in vivo*. HeLa-VC-luc and HeLa-KMT2B-luc cells (expressing the comparable levels of luciferase) were injected into nude mice by the tail vein. The bone metastatic nodules were visualized by IVIS and further confirmed by micro-computed tomography (**Fig. 5A**). Interestingly, it showed that KMT2B overexpression significantly increased the incidence of bone metastasis (83.3% *vs.* 16.7%, *p* < 0.05) rather than lung metastasis (0% *vs.* 0%) of Hela cells (**Table S4**). Meanwhile, the average number of bone metastatic nodules in KMT2B-overexpressing group is remarkable greater than that of control mice (1.5 *vs.* 0.17) (*p* < 0.05, **Fig. 5B**). These results suggested that KMT2B facilitated the CC cells colonization in the bone marrow. To confirm the influence of KMT2B on intraosseous growth of CC cells, we constructed a xenograft model using intra-tibia tumor cell implantation. Fourteen days after intramedullary injection of tumor cells, the animals were checked for quantitatively evaluating luciferase bioluminescence intensity by IVIS every week to monitor tumor growth. The results showed that the mice injected with HeLa-KMT2B-luc cells had a significantly higher bioluminescence intensity (*p* < 0.05,** Fig. 5C**). The myelogenous CC xenografts in both groups were confirmed by histopathological examination (**Fig. 5D**). Moreover, there were significantly more Ki67 positive cells in HeLa-KMT2B group than the control group (*p* < 0.05, **Fig. 5E**), indicating that KMT2B promoted HeLa cell growth in the bone marrow microenvironment.

Collectively, these findings demonstrated that KMT2B promoted CC bone metastasis and angiogenesis *in vivo*.

### KMT2B promotes CC cell migration and invasion through activating PI3K/AKT signaling pathway

**Figure 6:**
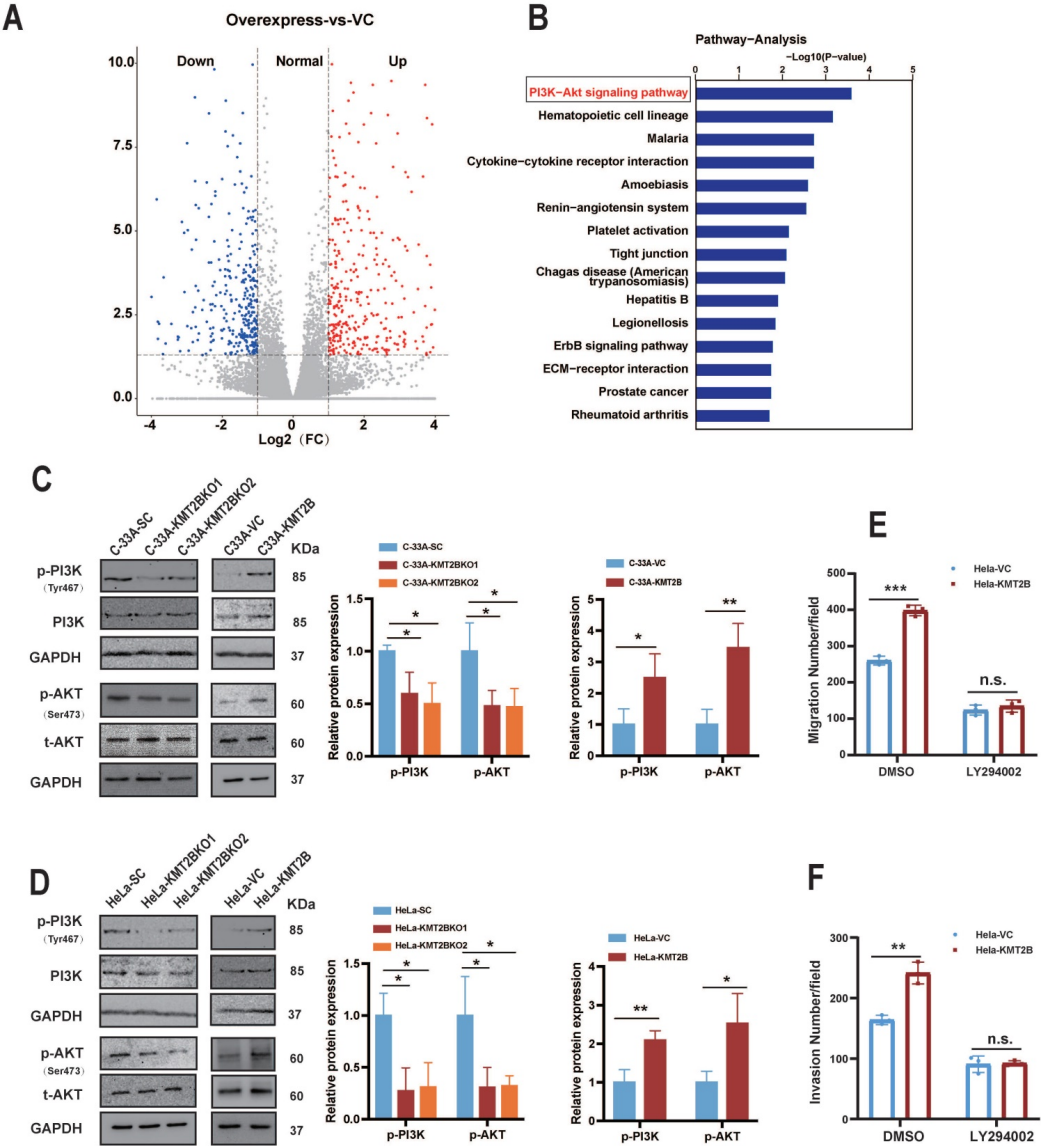
** KMT2B promotes CC cell migration and invasion through activating PI3K/AKT pathway. (A)** The volcano graph shows the distribution of differentially expressed genes from RNA-seq data between HeLa-VC and HeLa-KMT2B groups. **(B)** The graph shows the top 15 KEGG enrichment pathways of KMT2B-mediated upregulated genes. (n = 4 for each group). **(C, D)** Western blotting detected the expression of p-PI3K, total PI3K, p-AKT, and total AKT in KMT2B-overexpressing and KMT2B-knockdown CC cells. The histograms at the right show the data of densitometric analyses. **(E)** HeLa-VC and HeLa-KMT2B cells with or without LY294002 treatment were cultured in non-coated chambers for 8 h. The cell migration was quantitated by counting the migrated cells. **(F)** HeLa-VC and HeLa-KMT2B cells with or without LY294002 treatment were cultured in Matrigel-coated chambers for 12 h. The cell invasion was quantitated by counting the invaded cells. Graph bars represent the mean ± SD (n = 3) All the experiments have been repeated at least once with similar results. *, *p* < 0.05, **, *p* < 0.01; ***,* p* < 0.001, *ns*, not significant.

To determine how KMT2B promotes the malignant behaviors of CC cells, we compared the whole genome mRNA expression profile of HeLa-VC and HeLa-KMT2B cells using RNA-seq. A distinct transcriptome difference was observed (**Fig. 6A**) with 176 genes significantly upregulated and 347 genes downregulated by KMT2B overexpression (fold change ≥ 2 or ≤ 0.5, *p* < 0.05). The KEGG pathway enrichment analysis revealed that the most significantly enriched pathways of upregulated genes was PI3K/AKT signaling pathway (*p* < 0.05, **Fig. 6B**). Furthermore, the levels of phosphorylated PI3K (p-PI3K) and phosphorylated AKT (p-AKT) was significantly increased in HeLa-KMT2B and C-33A-KMT2B cells, and significantly decreased in HeLa-KMT2BKO and C33A-KMT2BKO cells (*p* < 0.01 or *p* < 0.05, **Fig. 6C & D**), indicating that KMT2B activated the PI3K/AKT signaling pathway in CC cells. Specific inhibition of PI3K activity by LY294002 markedly impeded the KMT2B-induced migration (*p* < 0.001, **Fig. 6E**) and invasion (*p* < 0.01, **Fig. 6F**) of HeLa cells, suggesting that the PI3K/AKT signaling pathway was required for KMT2B-mediated CC cell migration and invasion.

### EGF is the key target gene of KMT2B and mediates the KMT2B-induced malignant behaviors of CC cells

**Figure 7:**
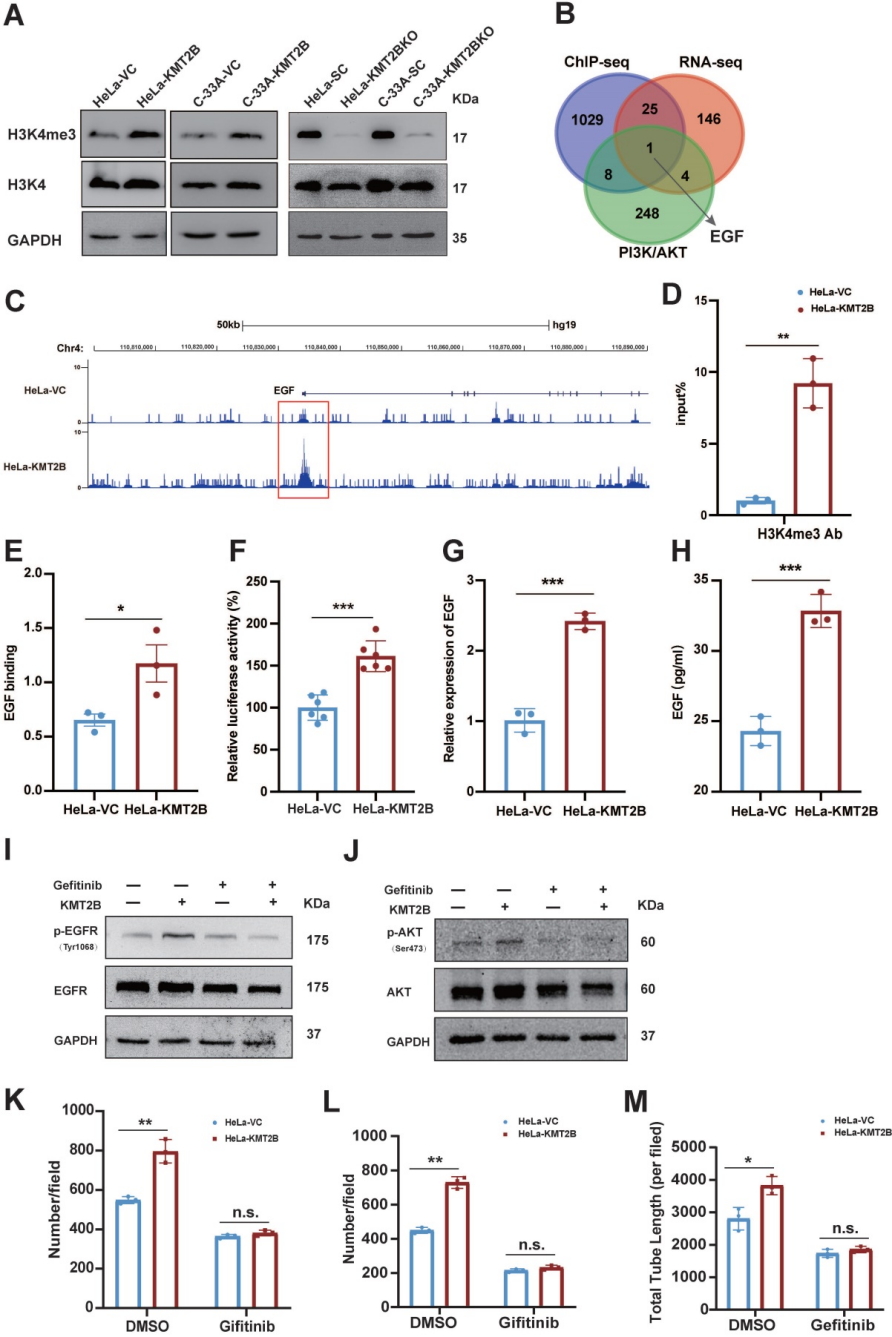
** EGF is a target gene of KMT2B in CC cells and mediates KMT2B-dependent PI3K/AKT activity. (A)** Effects of KMT2B overexpressing on the global levels of histone H3K4 tri-methylation using western blotting analysis. **(B)** Intersection analysis of differential genes in RNA-seq, CHIP-seq and PI3K/AKT signaling pathway related genes. **(C)** Visualization of the H3K4me3 enrichment in the promoter region of EGF gene HeLa-VC and HeLa-KMT2B cells. **(D)** H3K4me3 CHIP-qPCR analysis was used to determine H3K4me3 level at EGF promoter region in HeLa-VC and HeLa-KMT2B cells. **(E)** ChIP-qPCR was used to verify the binding of KMT2B at the EGF promoter region in HeLa-VC and HeLa-KMT2B cells. **(F)** Luciferase reporter genes driven by the EGF promoter (pGL3-EGF) were co-transfected with pRL-TK into HeLa-VC and HeLa-KMT2B cells, and luciferase signals were measured after 48 h. The relative luciferase activity value in HeLa-VC cells was set to 100%. **(F)** qPCR assays confirmed the difference in expression of EGF gene between HeLa-VC and HeLa-KMT2B cells at mRNA level. **(G)** ELISA assays confirmed the difference in expression of EGF gene between HeLa-VC and HeLa-KMT2B cells at protein level. **(H, I)** Western blotting analysis was used to detect the expression of p-EGFR and p-AKT after treatment of gefitinib in HeLa-VC and HeLa-KMT2B cells. **(J)** HeLa-VC and HeLa-KMT2B cells with or without gefitinib treatment were cultured in non-coated chambers for 8 h. The cell migration was quantitated by counting the migrated cells. **(K)** HeLa-VC and HeLa-KMT2B cells with or without gefitinib treatment were cultured in Matrigel-coated chambers for 12 h. The cell invasion was quantitated by counting the invaded cells. **(L)** Quantitated the Total length of tubes by Image Pro Plus software after incubating in HeLa-VC and HeLa-KMT2B condition medium with or without gefitinib treatment for 4 h. Data represents mean ± SD (n = 3). All the experiments have been repeated at least once with similar results. *, *p* < 0.05, **, *p* < 0.01; ***, *p* < 0.001, *ns*, not significant.

KMT2B is responsible for tri-methylation of H3K4 11. As expected, H3K4me3 level were significantly increased in the KMT2B-overexpressing CC cells and decreased in the KMT2B-knockdown CC cells (**Fig. 7A**). Since KMT2B upregulates genes expression by catalyzing tri-methylation of lysine 4 on histone 3 at the promoter regions of target genes 15, using purified anti-H3K4me3 antibody, we performed CHIP-seq analysis to map the genome-wide of H3K4me3 binding sites in HeLa-VC and HeLa-KMT2B cells. Comparative analysis of H3K4me3 peak differential enrichment regions on the whole genome between HeLa-VC and HeLa-KMT2B cells showed that total 2571 gene promoter regions were differentially enriched with H3K4me3 peak (*p* < 0.001). (**Table S5**). There were 1063 gene promoter regions with increased H3K4me3 level (fold change ≥ 3, *p* < 0.001), among which 26 genes showed transcriptional up-regulation by KMT2B as revealed by our RNA-seq. Then we investigated the overlapping gene set between these 24 upregulated genes and PI3K/AKT signaling pathway associated genes. We found that only one target gene EGF in the set, which not only showed increased H3K4me3 peak at promoter region but also be related to PI3K/AKT signaling pathway (**Fig. 7B-C**). To confirm the ChIP-seq results, we measured the levels of H3K4me3 at EGF promoter region using anti-H3K4me3 antibody in ChIP-qPCR experiment. In HeLa-KMT2B cells, increased H3K4me3 levels were confirmed in the promoter region of EGF (*p* < 0.01, **Fig. 7D**). Then, ChIP-qPCR analysis was performed using anti-KMT2B antibody to determine whether KMT2B bound to the EGF promoter region. The results showed that KMT2B-overexpression resulted in a significant increase of KMT2B binding at EGF promoter region (*p* < 0.05, **Fig. 7E**). Also, the results from luciferase report assay showed that KMT2B overexpression significantly enhanced the luciferase activity driven by EGF promoter fragment (*p* < 0.001, **Fig. 7F**). These findings indicated that KMT2B physically bound to EGF promoter region and enhanced the transcriptional activity of EGF in CC cells. Further, to evaluated whether KMT2B upregulated the expression of EGF, qPCR assay was performed to detect the mRNA level of EGF in HeLa-VC and HeLa-KMT2B cells. Consistent with RNA-seq results, the qPCR result showed that KMT2B overexpression markedly promoted the transcription of EGF (*p* < 0.001, **Fig. 7G**). Moreover, ELISA assay showed that KMT2B significantly promoted EGF protein level in HeLa-KMT2B cells (*p* < 0.001, **Fig. 7H**). These data demonstrated that EGF was a key target gene of KMT2B and was upregulated in KMT2B overexpression HeLa cells.

EGF is an upstream regulator of PI3K/AKT signaling pathway and plays an important role in tumor metastasis 43. In our study, we observed that EGFR inhibitor gefitinib abrogated the KMT2B-induced AKT phosphorylation in HeLa cells by western blotting assay (**Fig. 7I-J**), suggesting that KMT2B activated PI3K/AKT signal pathway through upregulated the expression of EGF. To evaluate the effect of EGF on KMT2B-mediated of CC cells migration and invasion, we treated HeLa-VC and HeLa-KMT2B cells with gefitinib. Transwell migration and invasion assays showed that gefitinib significantly hindered the ability of migration (*p* < 0.01,** Fig. 7K**) and invasion (*p* < 0.01,** Fig. 7L**) in HeLa-KMT2B cells. The result showed that KMT2B promoted CC cells migration and invasion through EGF/PI3K/AKT axis. In addition, we also treated HUVECs with gefitinib to investigate whether EGF also mediated the KMT2B-ibduced enhancement of tube formation. It showed that gefitinib remarkably abolished the KMT2B-induced HUVECs tube formation capacity (*p* < 0.05, **Fig. 7M**).

Collectively, KMT2B promoted the CC cells migration, invasion and pro-angiogenic effect by upregulated the expression of EGF.

## Discussion

In recent years, KMT2B has attracted increasing attention because of its important role in depositing H3K4me3 at promoters that active gene expression, rationalizing its widespread involvement in various pathological processes 44, 45. The KMT2B-H3K4me3 axis has been involved in the occurrence and progression of a variety of cancers, such as breast cancer, colon cancer, and liver cancer 20, 21, 46. In the context of CC, a previous study showed that the expression of H3K4me3 was detectable in almost all CC tissues (96.8%) and enhanced expression of H3K4me3 was correlated with poor prognosis of CC patients, suggesting an important role of H3K4me3-mediated epigenetic regulation in CC 47. However, no study has investigated the role of KMT2B in CC. The present study, for the first time, focused on the role of KMT2B in CC. The results showed that KMT2B levels were higher in CC tissues than normal cervical tissues. Importantly, we observed a strong association between high tumoral KMT2B expression and poor survival outcomes of CC patients. A number of prognostic factors for recurrence and survival of CC have been identified, including age, stage, histological type, grade, lymph node involvement and location 48. However, molecular markers responsible for cervical cancer prognosis are insufficient. The present study showed that KMT2B may be an effective prognostic molecular marker in outcome prediction for CC patients. Nevertheless, whether KMT2B is an independent risk factor of CC requires further investigation in a larger clinical cohort.

Most previous studies have described KMT2B as a positive regulator of cell proliferation in colon cancer and breast cancer 20, 21. However, we found that KMT2B could positively regulate the migration and invasion rather than the proliferation of CC cells, suggesting that the effect of KMT2B might be cell-type or context dependent. There are a few studies showing the role of KMT2B in cancer metastasis. A most recent study reported the involvement of KMT2B in the brain metastasis of lung adenocarcinoma 49. The current study supports the role of KMT2B in cancer metastasis and indicates that KMT2B facilitates the bone metastasis of CC. Bone is one of the most common metastatic sites of CC 50, 51. The CC patients with bone metastasis have an extremely poor prognosis 2, 52 and the median survival is less than 6 months in the absence of appropriate therapy 53. However, the underlying molecular mechanisms for the affinity of CC cells towards skeletal bones are still not completely understood. We found that KMT2B-overexpression CC cells not only had a strong predilection for bone spreading to limb but also exhibited more rapidly growth in bone marrow compared to control group. Further in-depth research focusing on KMT2B may provide new insights into the molecular mechanisms and offer potential novel target for clinical interventions of CC metastasis.

The present study also provided evidence demonstrating KMT2B as a novel pro-angiogenic factor in CC. Solid tumor growth can be separated into two stages, avascular and vascular. Avascular is the early stage of tumor growth, in which most solid tumors seem capable of living by simple diffusion until they reach a diameter of a few millimeter 54. However, tumor growth begins to accelerate in vascular stage 54. The rapid growth of tumor in vascular stage requires enormous nutrients via blood vessels 55. In our study, KMT2B significantly increased tumor progression in the late stage (after 14 days) compared to the control group, suggesting that KMT2B might promote tumor growth in vascular stage. Importantly, we observed that CD31-marked vessels were more abundant in KMT2B overexpressed tumors. These results indicated that KMT2B acts as pro-angiogenic gene in CC cells and facilitates tumor growth by enhancing angiogenesis. Tumor angiogenesis is an independent prognostic parameter of disease-free survival in patients with CC 56. Anti-angiogenic therapy has been proven to be an effective treatment strategy for advanced or recurrent CC patients 57. Meanwhile, migratory and invasive behaviors of CC cells also contribute to the tumor growth in the late stage. Together, based on our present researches, KMT2B-targeted therapy may be beneficial to CC patients with high KMT2B expression by inhibiting local tumor growth via impairing angiogenesis.

We identified EGF for the first time as a target gene which is regulated by KMT2B. KMT2B is responsible for the deposition of H3K4me3 at gene promoters to upregulate the target genes transcription. For instance, in non-small cell lung cancers, KMT2B increased the c-Myc transcription 58. In colon cancers, KMT2B maintained the H3K4me3 status at the LGR5 promoter to increase the transcription of LGR5 21. Here, we found that in CC, KMT2B overexpression increased H3K4me3 levels at the EGF promoter region and promoted EGF genes transcription. EGF regulates cancer metastasis and angiogenesis through multiple mechanisms. Autocrine production of EGF by cancer cells contributes to cancer cells migration and invasion. EGF through paracrine stimulation of normal endothelial promotes tumor neovascularization 59. Consistently, our research showed that KMT2B-induced EGF promoted the CC cells migration and invasion by activating PI3K/AKT signaling. Meanwhile, EGF also enhanced the angiogenesis in CC. Thus, we characterized EGF as a key downstream pathway factor to effect KMT2B-mediated malignant behaviors of CC. Targeting against EGFR might be useful to improve therapeutic efficiency in CC patients with high KMT2B expression.

**Figure 8:**
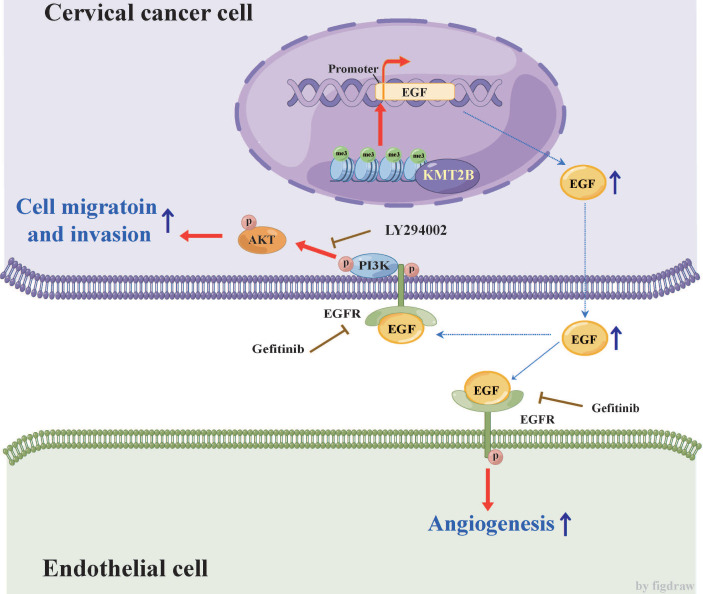
** Schematic diagram illustrating the proposed mechanisms of the effects of KMT2B on CC.** In CC cells, KMT2B promotes the expression of EGF by maintaining the status of H3K4 tri-methylation at its promoter region. CC cell-derived EGF activates EGFR and promotes the angiogenesis of endothelial cells in a paracrine manner. Meanwhile, increased EGF expression enhances the migration and invasion of CC cells in an autocrine manner by activating EGFR/PI3K/AKT pathway.

In conclusion, the present study demonstrated that KMT2B is upregulated in CC tissues or cells and associated with poor prognosis. It also showed that KMT2B promoted CC cells metastasis and angiogenesis both *in vitro* and* in vivo* by upregulated EGF (see **Fig. 8** for the detailed mechanism). These results indicated a dual role of KMT2B in CC progression and suggested that KMT2B and EGF has potential therapeutic value in the treatment of CC.

## Supplementary Material

Supplementary figure and tables.
