# Supplementary material for: Histone Methyltransferase KMT2B Promotes Metastasis and Angiogenesis of Cervical Cancer by Upregulating EGF Expression
Source: Int J Biol Sci. 2023 Jan 1;19(1):34–49. doi: 10.7150/ijbs.72381 (PMC9760441; doi:10.7150/ijbs.72381)
Supplement: Supplementary file 1 — Supplementary figure and tables. [file ijbsv19p0034s1.pdf]

Histone Methyltransferase KMT2B Promotes Metastasis and Angiogenesis of Cervical Cancer by Upregulating EGF Expression

Dan Zhao, Hui Yuan, Yuan Fang, Jian Gao, Huimin Li, Mengge Li, Hui Cong, Chenglin Zhang, Yiyi Liang, Jin Li, Hancao Yang, Ming Yao, Min Du, Hong Tu, Yu Gan

Supplementary Data

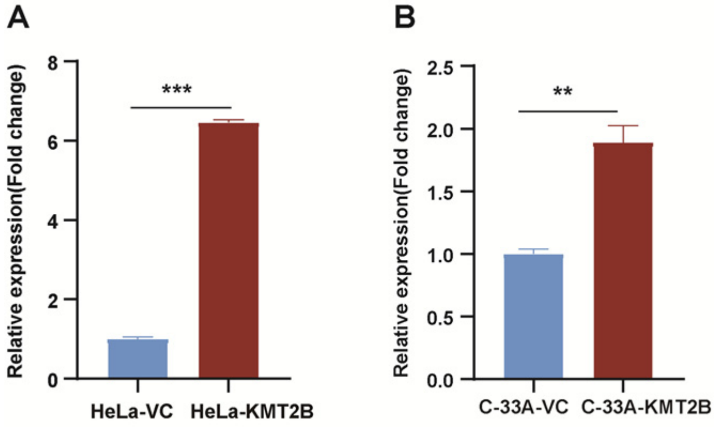

Figure S1. Quantitative real-time PCR analysis of the KMT2B mRNA expression level in KMT2B overexpression CC cells. (A) The expression of KMT2B in HeLa-VC and HeLa-KMT2B cells. (B) The expression of KMT2B in C-33A-VC and C-33A-KMT2B cells. *$P < 0.05$, **$P < 0.01$, *** $P < 0.001$.

Table S1. Clinicopathologic features of CC cases

Tissue No.	Gender	Age	Histological grade	Clinical stage	Pathological typing
J03A0274	Female	34	G2	II	Squamous cell carcinoma
J03A0275	Female	44	G2	II	Squamous cell carcinoma
J03A0276	Female	26	G2	II	Squamous cell carcinoma
J03A0277	Female	39	G2	II	Squamous cell carcinoma
J03A0278	Female	40	G2	II	Squamous cell carcinoma
J03A0279	Female	38	G2	II	Squamous cell carcinoma
J03A0280	Female	49	G2	II	Squamous cell carcinoma
J03A0281	Female	49	G2	II	Squamous cell carcinoma
J03A0282	Female	46	G2	II	Squamous cell carcinoma
J03A0283	Female	47	G2	II	Squamous cell carcinoma
J03A0284	Female	46	G2	II	Squamous cell carcinoma
J03A0285	Female	36	G2	II	Squamous cell carcinoma
J03A0286	Female	38	G2	II	Squamous cell carcinoma
J03A0287	Female	38	G2	II	Squamous cell carcinoma
J03A0288	Female	35	G2	II	Squamous cell carcinoma
J03A0289	Female	58	G2	II	Squamous cell carcinoma
J03A0290	Female	41	G2	II	Squamous cell carcinoma

J03A0291	Female	41	G2	II	Squamous cell carcinoma
J03A0292	Female	33	G2	II	Squamous cell carcinoma
J03A0293	Female	39	G2	II	Squamous cell carcinoma
J03A0214	Female	Adult	G1	II	Squamous cell carcinoma
J03A0218	Female	Adult	G3	II	Squamous cell carcinoma
J03A0220	Female	Adult	G1	II	Squamous cell carcinoma
J03A0235	Female	Adult	G2	II	Squamous cell carcinoma
J03A0242	Female	Adult	G2	II	Squamous cell carcinoma
J03A0027	Female	36	G1	II	Squamous cell carcinoma
J03A0033	Female	45	G2	II	Squamous cell carcinoma
J03A0045	Female	42	G2	II	Squamous cell carcinoma
J03A0062	Female	46	G2	II	Squamous cell carcinoma
J03A0136	Female	42	G2	II	Squamous cell carcinoma
J03A0141	Female	48	G3	II	Squamous cell carcinoma

Table S2. sgRNA sequence for *KMT2B* gene knockout

Seq No.	Forward (5’-3’)	Reverse (5’-3’)
sgRNA1	caccGTGCGGGTAGCTCTGCGGCG	aaacCGCCGCAGAGCTACCCGCAC
sgRNA2	caccGAAAGAGTGCGGGTAGCTCTG	aaacCAGAGCTACCCGCACTCTTTC

Note: cacc and aaac in lower case are overhangs for ligation into the pair of *Bbs*I sites in pSpCas9(BB)-2A-GFP (PX458) vector.

Table S3. Primers used in quantitative real-time PCR and ChIP-PCR

Target	Primer Sequence (5’-3’)
KMT2B	Forward: CCGAGTCGAGGCTGCGTG
	Reverse: CTCGCTGGGATCGGAGCG
EGF	Forward: TGTCCACGCAATGTGTCTGAA
	Reverse: CATTATCGGGTGAGGAACAACC
GAPDH	Forward: AGAAGGCTGGGGCTCATTTG
	Reverse: AGGGGCCATCCACAGTCTTC
EGF promoter	Forward: TTCACCATGAGCACCTCCAC
	Reverse: GCTCTGGCTGACTTCACTGT

Table S4. Number of animal with metastasis in tail-vein injection mouse model

	HeLa-VC	HeLa-KMT2B	*P* value
Bone metastasis	1/6 (16.7%)	5/6 (83.3%)	0.018
Lung metastasis	0/6 (0%)	0/6 (0%)	NA

Table S5. Genes showing transcriptional up-regulation and increased H3K4me3 peak at promoter region in KMT2B-overexpressing HeLa cells

Gene	RNA-seq				ChIP-seq				
	HeLa-KMT2B FPKM (average)	HeLa-VC FPKM (average)	Log2FC (HeLa-KMT2B /HeLa-VC)	*P*	HeLa-KMT2B Tags	HeLa-VC Tags	Fold change	Distance to TSS	*P*
GPR162	19.75	1.75	3.53	3.32E-07	150.6	25	6.02	-10	1.43E-64
MAGEB18	6	0	5.06	1.23E-04	29.3	1	29.3	-27	4.30E-32
EGF	33.25	6.75	2.37	7.93E-04	15	5	3	128	6.98E-04
A1CF	14	0.5	4.84	1.72E-05	24.03	0.5	48.53	199	5.83E-32
HMOX2	74.75	12.25	2.74	8.61E-04	12.8	2	6.42	-690	1.36E-06
NPPB	58	2	5.01	2.67E-03	59.9	11	5.45	-338	4.08E-24
CHFR	44.75	2	4.51	9.64E-11	38.5	3	12.85	-67	1.39E-28
SULT1C2	32.75	5	2.71	1.91E-06	17.8	1	17.8	-241	1.09E-15
IL31RA	207.5	22	3.28	2.89E-32	43.5	0.5	87.07	-3	1.14E-66
MAGEA9	10.75	1.25	3.16	1.56E-03	22.8	6	3.81	-643	3.91E-07
PCLO	467.25	13	5.26	4.42E-26	52.1	7	7.44	-277	1.15E-27

MAGEB6	24.75	1.5	4.08	1.77E-07	27.1	2	13.56	115	1.80E-21
CARD6	61.5	4.75	3.72	1.45E-12	32.1	6	5.35	173	9.15E-14
ANK2	13.75	1.75	2.97	1.42E-04	93.5	7	13.6	197	3.34E-69
SULT2A1	12.5	2	2.65	8.94E-04	16.4	2	8.2	-56	4.80E-10
CFLAR-AS1	15.75	22.75	2.64	3.33E-03	62.8	11	5.71	-611	2.55E-10
DPPA2	7.25	0.25	4.60	2.68E-04	13.6	4	3.39	-257	2.74E-04
EDIL3	46.25	2.5	4.18	1.20E-07	107.1	32	3.35	170	1.65E-25
IKZF2	13.25	1.75	2.93	1.62E-03	119.2	26	4.58	-924	2.82E-40
GBP1	49.75	1.5	5.10	9.49E-08	21.4	2	10.71	142	6.11E-15
SPTB	310	61	2.38	8.06E-32	22.1	7	3.16	-373	4.53E-06
CYB5R2	28.5	5	2.61	3.78E-04	37.1	3	12.37	-2920	1.77E-27
ARHGEF25	88.25	19.25	2.25	1.43E-12	16.4	1	16.4	-671	1.87E-14
LOC101926940	47.25	9	2.42	3.73E-06	30	4	7.49	-1623	6.8E-16
LOC729987	7.75	0.25	4.71	1.18E-03	80.6	4	20.16	45	3.93E-73
LINC00664	10.25	0	5.87	1.24E-05	41.4	11	3.76	-617	3.36E-12
